# Current and Future Prospects for Epigenetic Biomarkers of Substance Use Disorders

**DOI:** 10.3390/genes6040991

**Published:** 2015-10-14

**Authors:** Allan M. Andersen, Meeshanthini V. Dogan, Steven R.H. Beach, Robert A. Philibert

**Affiliations:** 1 Department of Psychiatry, University of Iowa, Iowa City, IA 52242, USA; E-Mails: allan-andersen@uiowa.edu (A.M.A.); meeshanthini-vijayendran@uiowa.edu (M.V.D.); 2 Department of Biomedical Engineering, University of Iowa, Iowa City, IA 52242, USA; 3 Department of Psychology, University of Georgia, Athens, GA 30602, USA; E-Mail: srhbeach@uga.edu; 4 Center for Family Research, University of Georgia, Athens, GA 30602, USA

**Keywords:** biomarkers, epigenetics, substance use disorders, addiction, smoking, tobacco, alcohol, cannabis, opioids, psychostimulants

## Abstract

Substance abuse has an enormous impact on economic and quality of life measures throughout the world. In more developed countries, overutilization of the most common forms of substances of abuse, alcohol and tobacco, is addressed primarily through prevention of substance use initiation and secondarily through the treatment of those with substance abuse or dependence. In general, these therapeutic approaches to substance abuse are deemed effective. However, there is a broad consensus that the development of additional tools to aid diagnosis, prioritize treatment selection and monitor treatment response could have substantial impact on the effectiveness of both substance use prevention and treatment. The recent demonstrations by a number of groups that substance use exposure is associated with robust changes in DNA methylation signatures of peripheral blood cells suggests the possibility that methylation assessments of blood or saliva could find broad clinical applications. In this article, we review recent progress in epigenetic approaches to substance use assessment with a particular emphasis on smoking (and alcohol) related applications. In addition, we highlight areas, such as the epigenetics of psychostimulant, opioid and cannabis abuse, which are markedly understudied and could benefit from intensified collaborative efforts to define epigenetic biomarkers of abuse and dependence.

## 1. Introduction

**Figure 1:**
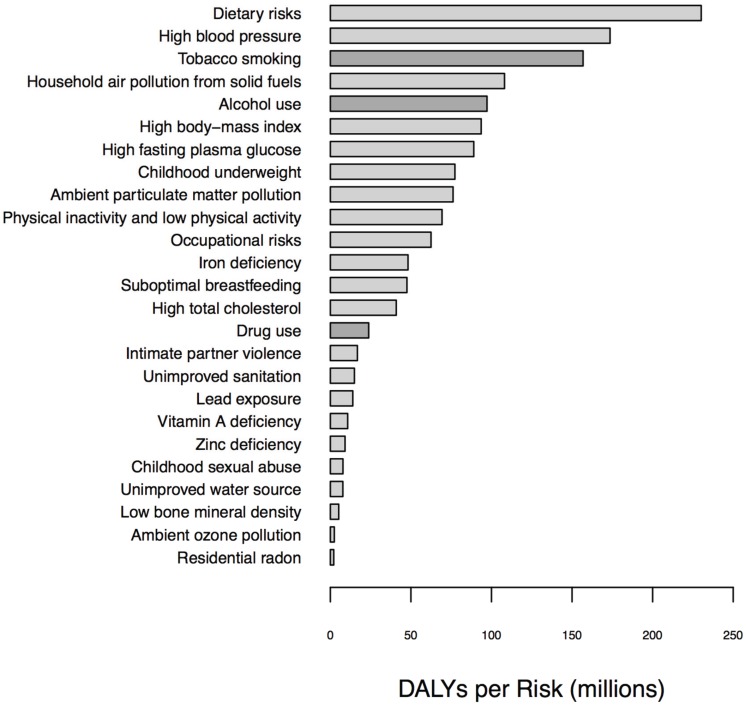
Global disability-adjusted life years (DALYs) annually by risk factor.

**Figure 2:**
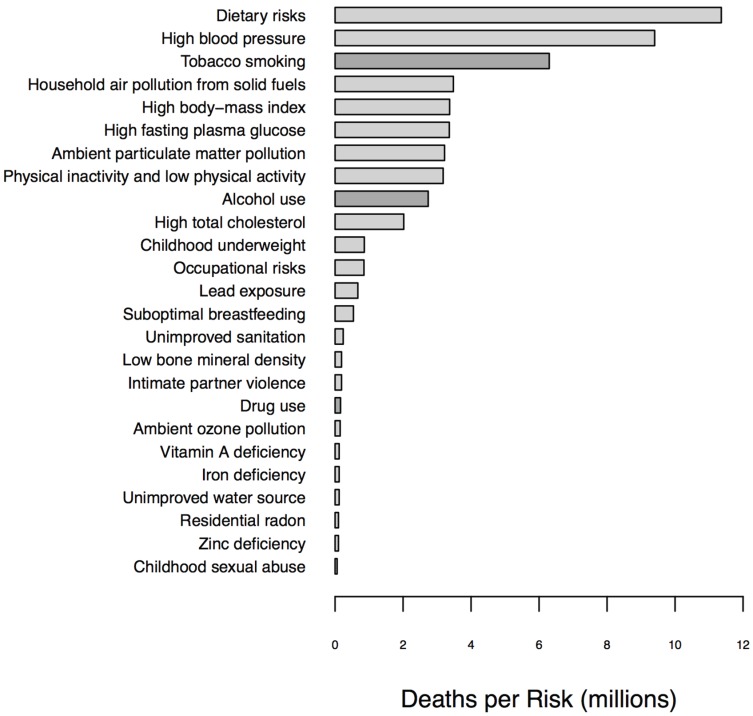
Global deaths annually by risk factor.

Substance use disorders are significant contributors to disability and mortality in the United States and across the globe (Figure 1 and Figure 2). According to the Institute for Health Metrics and Education, tobacco smoking, alcohol use, and illicit drug use caused approximately 10.2 million deaths globally in 2010, with the majority being due to smoking (7 million deaths) and alcohol (3 million deaths) use [1]. In the United States, it is estimated that approximately 70 million individuals use tobacco products, 75 million individuals engage in heavy or binge drinking, and 22 million individuals aged 12 or over engage in some illicit drug use on a monthly basis. Over 400,000 premature deaths yearly are due to medical disorders directly attributable to the effects of smoking, including heart disease, chronic obstructive pulmonary disease, and cancer. Similarly, each year, 100,000 premature deaths are attributable to the downstream effects of alcohol, including deaths from both accidents and chronic illness such as cirrhosis [2].

The economic burden of substance use is equally substantial. In the United States, both smoking and alcohol use result in over $200 billion dollars of lost wages, treatment and other economic costs annually [3]. The criminal justice system is significantly burdened by the costs of incarceration of those with substance use disorders. Among these, approximately one million individuals are addicted to cocaine and nearly 1.5 million abuse or are dependent on heroin or prescription opiates [4].

There is hope on the horizon for the relief of these scourges, but there are barriers to treatment and prevention implementation. Evidence-based treatments for substance use disorders include both pharmacologic and behavioral components. Despite the existence of these effective treatments, the lack of ability to reliably detect problematic substance use hinders clinical efforts to direct patients toward appropriate care. Additionally, after identification of the disorder and the initiation of treatment, clinicians often have difficulty monitoring the patient’s response to treatment and success in maintaining abstinence. This inability to detect relapse can lead to delays in making appropriate treatment adjustments to improve patient outcomes.

Identifying substance use disorders in primary care contexts is difficult for multiple reasons. Like other psychiatric disorders, substance use disorders are typically not associated with objective physical findings, and laboratory test results alone are not sufficient to make a diagnosis or monitor response to treatment. Furthermore, in contrast to most other psychiatric disorders, patient with substance use disorders often attempt to conceal rather than reveal true patterns of use, both in primary care and specialty settings. Even in acute clinical situations where evidence of a substance use disorder is readily apparent, such as hospitalization for alcohol withdrawal, users may under-report their overall pattern of use, particularly with respect to comorbid illicit substance use [5]. As a result, for each individual identified as needing treatment for a substance use disorder, a large number of other users never come to clinical attention. This is particularly concerning in child and adolescent populations, where there is the greatest potential for a change in an individual’s long-term psychosocial and physical health trajectory as well as for clinical outcomes.

The limitations of current technology in detecting smoking, alcohol use, and illicit substance use are well documented and further contribute to difficulty in identifying patients in need of treatment. Cotinine, a metabolite of nicotine, is detectable in blood and urine, but the window for detection is only 48 h [6,7]. Exhaled carbon monoxide has an even shorter window of detection with a half-life of 4.5 h [7,8]. Similarly, there are serious deficiencies in current algorithms to detect problematic alcohol use, including biomarkers such as serum aspartate aminotransferase, carbohydrate-deficient transferrin, and gamma-glutamyl transferase, which are limited by invasiveness of testing (blood *versus* urine), poor sensitivity and specificity, and high cost [9]. For other substances, screening generally relies on urine testing, for which urine immunoassays exist but have similar limitations, including a number of potential false-positives and false-negatives [10]. In the case of a positive result on urine immunoassay, which is currently recommended as the first-line screening test for illicit substances [10], confirmatory testing can be done using gas chromatography/mass spectrometry (GC/MS), which is more accurate but substantially more expensive and time consuming [11]. Also, limiting some practical applications is relatively slow detection. With the exception of heavy, chronic marijuana use, the timeframe for detection of most illicit substances by either immunoassay or GC/MS is on the order of a few days [10].

Despite clear evidence of the public health burden of substance use disorders, difficulty in detecting these across the lifespan is likely to continue given the current legal and social milieu. Illicit substance use carries legal and occupational risks, which creates tension for patients who are motivated to continue using despite these risks due to addiction. Many require routine urine drug testing, such as those in the transportation industry or those requiring the use of heavy equipment, and positive tests for illicit substances may be grounds for dismissal. In addition, although tobacco smoking is legal, many organizations, particularly large organizations tasked with providing healthcare for employees, have begun to routinely monitor for tobacco use in order to incentivize cessation. Although beneficial from the perspective of motivating cessation, such policies also create incentives to avoid detection despite ongoing use, impairing clinicians’ ability to intervene appropriately. Social stigma provides an additional incentive to conceal substance use, and in the case of adolescents, fear of disciplinary intervention does as well. Thus, although individuals as well as private and public organizations all share an interest in improving current technology to accurately detect substance use patterns, improvement in detection is unlikely to improve significantly without the development of improved means of detecting these problems.

### 1.1. Biomarkers

In order to meet the challenges posed by substance use disorders, it is essential that we develop improved biomarkers to allow detection of these disorders. As reviewed by Mikeska and Craig [12], a biomarker is an objectively measurable characteristics of an organism that allows monitoring of a biological process related to normal physiology, pathophysiology, or disease. Clinically relevant biomarkers may serve a variety of purposes, such as indicating disease latency, onset, stage, response to treatment, may serve as a surrogate endpoint for intervention, or may help stratify individuals according to risk or prognosis. For maximum public health impact, biomarkers should be accurately measurable across individuals and populations. Ideal biomarkers will have high sensitivity and specificity, a high area under the curve (AUC) in a receiver-operator characteristic (ROC) analysis, and a high positive predictive value (PPV).

Put in the language of healthcare economics, in order to be clinically useful, biomarkers must balance the competing vertices of the “iron triangle of health care”: cost, quality, and access [13]. That is, firstly, biomarkers should be affordable enough to be used effectively in the population and disease in question. Secondly, biomarkers should have adequate sensitivity and specificity to facilitate prudent clinical judgments in the population and disease in question. And thirdly, biomarkers should have broad applicability across individuals and populations whenever possible, and be from an easily obtainable tissue.

Hemoglobin A1c, for example, may be considered a nearly ideal biomarker based on the above criteria [14]. It is relatively inexpensive and easy to assay, relevant across individuals and populations, and clinically useful within an individual across time in terms of both disease progression and treatment response. It also helps stratify individuals’ risks for diabetes-associated health risks and outcomes.

In contrast, in the domain of psychiatry, clinically relevant biomarkers are scarce. For a few uncommon, Mendelian disorders such as Fragile X syndrome and Huntington’s disease, genetic testing is available. Recent work by Guintivano and colleagues has demonstrated that some psychiatric conditions such as suicidality may have emerging biomarkers, but such findings are not yet well-validated [15]. Similarly, pharmacogenomics, the study of differential response to medication treatment due to inborn differences in metabolism, is a developing field with potential relevance in psychiatry that has yet to prove its clinical relevance [16].

For substance use disorders, new biomarkers that improve on existing technology are highly desirable. But in addition to the general factors listed above, researchers must consider additional factors specific to substance use disorders when considering whether potential biomarkers will improve on current technology. First, given that substance use is often intermittent, biomarkers must offer adequate stability and persistence to allow detection at a future time point. Lack of persistence over time is a considerable weakness in current biomarkers for substance use (see Table 1). Next, specificity for the type of exposure is essential. For example, given frequent comorbid patterns of substance use, distinguishing between new-onset cannabis use in the setting of ongoing tobacco smoking may be needed. Third, specificity with respect to cumulative exposure is important, as for many substances such as tobacco and alcohol, adverse outcomes are most strongly related to cumulative exposure. Fourth, the ability to detect initial cessation of use and ongoing abstinence is essential in implementing appropriate monitoring and assessing response to treatment. Fifth, specificity with respect to the exposure window is an important consideration for the development of the ability to detect specific kinds of exposure such as prenatal exposure.

**Table 1:** Detection time windows and false positives for commonly used substances.

Drug	Detection Time (Urine)	False Positives
Alcohol		
- Ethanol	Less than 12 h	No
- Ethyl glucuronide (metabolite)	5 days	No
Amphetamine/Methamphetamine	2–3 days	Amantadine, bupropion, chlorpromazine, desipramine, fluoxetine, labetalol, methylphenidate, phentermine, phenylephrine, phenylpropanolamine, promethazine, pseudoephedrine, ranitidine, thioridazine, trazodone
Cocaine		Topical anesthetics containing cocaine
- Occasional use	2–3 days	
- Heavy use	Up to 8 days	
Cannabis		Dronabinol, ibuprofen, naproxen, sulindac, proton pump inhibitors
- Single use	3 days	
- Less than daily	Up to 1 week	
- Daily	1 to 2 weeks	
- Daily, heavy use	>30 days	
Opioids		Dextromethorphan, diphenhydramine, fluoroquinolones, poppy seeds, quinine, rifampin, verapamil (methadone assays only)
- Codeine	2 days	
- Heroin (morphine)	2 days	
- Hydromorphone	2–4 days	
- Methadone	3 days	
- Morphine	2–3 days	
- Oxycodone	2–4 days	
Tobacco	Up to 1 week	Nicotine replacement therapy, nicotine vaporizers

Note: Data from references [10,11,17].

Lastly, unlike other psychiatric disorders, substance use disorders can also be conceptualized more broadly as a type of exposure. Although substance use disorders are defined behaviorally as a user’s repetitive self-administration of substances despite negative consequences and/or resultant physiological dependence, biomarkers indicating disease or risks to health may be present in both the user and others inadvertently exposed through the same actions. Given that non-voluntary exposures such as prenatal alcohol exposure or secondhand smoke exposure also carry significant health and developmental consequences [18], detection of these kinds of exposures is therefore an additional public health priority, and merits additional consideration of the similarities and differences of biomarkers for various substance exposures in different developmental or environmental contexts.

### 1.2. Epigenetic Biomarkers

Epigenetic biomarkers have the potential to address these critical issues. Epigenetics is the study of potentially heritable marks that provide structural and regulatory functions to the genome, but are distinct from changes in the base pair sequence of the genome [19]. Epigenetic marks include DNA methylation at CpG residues, histone tail modifications, small non-coding RNAs, and open *versus* closed chromatin packing. The basic concepts of epigenetics have been reviewed previously [12,18,20] and will not be discussed in detail here. Functionally, epigenetic changes affect the expression of genes, as measured by RNA and protein production, which in turn may affect cellular structure and function, which in turn may in turn lead to changes in higher level phenomena such as behavior. With respect to drug taking and drug seeking behavior, preclinical literature has demonstrated that changes in the expression of genes such as BDNF [21] and OPRM1 [22,23] alter the reinforcement properties of drugs such as alcohol, cocaine and heroin. Thus, in addition to having utility as biomarkers, epigenetic changes and resultant changes in gene expression can also contribute to our mechanistic understanding of addictions.

Among epigenetic marks, DNA methylation changes are most likely to develop as ideal substance use disorder biomarkers. As reviewed by Ladd-Acosta, there is a broad evidence base supporting DNA methylation signatures as biomarkers of exposure that are likely to translate well to clinical practice [18]. Methylation signatures for different types of environmental exposures (including smoking) have been shown to exhibit temporal stability, specificity with respect to type of exposure, timing of exposure, cumulative dose and cessation time. Equally important, current DNA methylation assays can be used with a variety of accessible tissue types and are becoming more accurate and inexpensive, thus addressing the key core areas of cost, quality, and access. In contrast, assays of histone tail modifications and RNAs are more expensive and technically difficult. Therefore, for reasons of both cost and quality, we will focus this review on DNA methylation biomarkers rather than histone or RNA biomarkers. Additionally, for reasons of specificity, we will focus on methylation patterns at specific CpGs as opposed to measurement of global methylation levels. Changes in in global DNA methylation, often measured via digestion and analysis of repetitive DNA motifs distributed throughout the genome such as Long Interspersed Element-1 (LINE-1), have been reported in cancer, but these techniques lack adequate specificity for use in substance use disorders.

Methylation at a given CpG residue is frequently reported as a percent methylated in a given sample, or beta value, with changes at the same location between individuals or groups often reported as a delta beta. Bisulfite pyrosequencing currently has a resolution of approximately 5%–10%, indicating that changes on this order of this magnitude may not be reliably detected. The resolution of array-based technologies such as Illumina’s HumanMethylation450 BeadChip is as precise as 1%–2% for comparison. A key issue for the development of clinically relevant biomarkers will be whether delta betas for a given association are of a sufficient magnitude to provide adequate sensitivity and specificity.

In this review, we will present evidence that recently identified DNA methylation loci show great promise as biomarkers for smoking, meeting the above criteria for translation to clinical use. Next, we will demonstrate that current evidence for methylation changes as biomarkers for alcohol use is much more limited but emerging, and finally that the evidence for other substances is still more limited and not at a point where translation to clinical use is feasible.

For translation to clinical applications, several areas are important to consider in the development of epigenetic biomarkers for substance use disorders. First, although substance use disorders are brain-based disorders, brain tissue is not accessible in routine clinical practice. Therefore, this review will focus primarily on findings in tissues that are more easily accessible, such as peripheral leukocytes and saliva samples, with other tissues discussed as relevant to highlight the generalizability of findings. Peripheral tissues do have important limitations with respect to generalizability to other tissues of interest such as the brain [24], but there is increasing evidence that many epigenetic changes found in peripheral leukocytes and transformed lymphoblasts also correspond to changes in brain cells [25].

Second, when dealing with peripheral blood cells, it is important to note that differences in cell mixture may confound differences in methylation patterns due to substance use. Since this problem has been recognized, it has become routine to control for such differences in cell composition through either direct cell counts or through the method of Houseman and colleagues [26], by which methylation patterns in a given sample can be analyzed to infer the original cell mixture distribution. However, it is important to note that the effect of these measures in improving signal in some disorders is debatable [27,28,29] and the recent discovery that many lymphoid cells do not display traditional cell specific markers used to develop the methylation data used in the Houseman technique suggests a need to refine these cell correction approaches [30].

Third, in order to address generalizability of findings, we will focus on research in human subjects rather than animal or *in vitro* models. Similarly, research in different ethnic groups, ages, and sexes will be included. The majority of research in substance use epigenetics has been in adults of European ancestry, but a few studies have included other ethnic groups and ages for comparison. In addition, when studies report differential findings by sex, these will be included in the review. These are particularly important aspects as studies have reported differences in global methylation in different sexes and ethnicities [31]. In addition to demographic aspects of study design, temporal aspects such as the measurement of changes in individuals or groups over time prospectively lend important additional aspects to clinical generalizability and may contribute to understanding of the underlying etiology of substance use disorders.

Lastly, we will comment on the status of specific biomarkers as to whether they are potential validated, replicated, candidate or proven clinical biomarkers, as defined by Mikeska and Craig [12]. When relevant, experiments that lend supporting evidence to methylation findings, such as changes in gene expression via RNA measurement, protein expression, and even DNA-protein complex binding, will also be included. The results of network and pathway analyses are also interesting because they lend support to the involvement of aspects of physiology such as the immune system in the pathogenesis of disease and their effect on measurable biomarkers. In addition, when supporting evidence of exposure, such as serum cotinine assays, is included, these data will be discussed.

## 2. Methods

PubMed searches were conducted with terms related to specific substance use disorders, epigenetics, and biomarkers. Resulting abstracts were reviewed and included for discussion if they appeared relevant to the specific topic of epigenetic biomarkers for substance use disorders. Citations mentioned within these publications were also reviewed for further inclusion in this review when appropriate.

## 3. Results

### 3.1. Smoking

In the process of smoking, thousands of chemicals, including carcinogenic polycyclic aromatic hydrocarbons (PAHs) and nitrosamines, are released into the human body [32]. Further downstream, nicotine exerts reinforcing effects in the CNS before being metabolized [33]. Interestingly, the presence of monoamine oxidase inhibitors (MAOIs) has also been demonstrated in tobacco [34], but the magnitude of their psychoactive effects and contribution to patterns of tobacco consumption remain unclear. Despite its widespread effects throughout the body, existing biomarkers for smoking have significant limitations. Exhaled carbon monoxide is detectable only for 3–4 h after smoking [6,7]. Cotinine, a metabolite of nicotine, can be assayed in serum or saliva, but can only be detected for approximately 48 h after last use [7], and the preferred method of detection, enzyme linked immunoassay (ELISA) is expensive and time consuming.

It should also be noted that the rise of e-cigarettes, which deliver nicotine via an atomized solution of polyethylene glycol, has the potential to confound the detection of smoking, as it will lead to a positive test for cotinine. Because the differences and similarities in health risks due to smoking cigarettes *versus* e-cigarettes are not yet known, it is even more important that new biomarkers be developed to accurately ascertain smoking status.

Research in epigenetic biomarkers for smoking encompasses three main waves of findings. First, many studies examined candidate genes such as *Monoamine Oxidase A* and *B* (*MAO-A*, *MAO-B*), often using bisulfite pyrosequencing techniques available in the 2000s to investigate the relationship between methylation at CpG islands and substance use disorders. Second, with the advent of array-based methylation detection technologies, a number of studies in varying populations, tissues, and sample sizes have been done. With the further development of arrays by Illumina and others, these studies have been able to expand from a limited number of CpG sites across the genome, generally focused in areas related to cancer, to a much broader range of sites. Third, based on the results of array-based studies, follow-up studies of promising loci have been done to more carefully delineate methylation patterns associated with smoking and investigate potential utility as biomarkers for smoking and related health risks. As it will be demonstrated in the rest of this review, this trajectory places smoking epigenetic biomarkers closest to translation into clinical practice, while other substance use disorders under study remain less developed.

Findings from the first wave of candidate gene methylation studies for smoking (approximately 2008–2012), generally using bisulfite pyrosequencing or mass spectrometry, established the existence of differences in methylation between cases and controls at the promoters of several candidate genes for smoking, including *MAO-A and MAO-B*. Two early studies by Philibert and colleagues [35,36] found that symptom counts for nicotine dependence were associated with decreased methylation at the MAO-A promoter, that genotype and sex-specific effects influenced methylation, and that changes in methylation pattern persisted over time after smoking cessation. There also did not appear to be a significant difference between methylation patterns in whole blood samples *versus* transformed lymphoblasts. Launay and colleagues [37] similarly found a decrease in MAO-B promoter methylation in peripheral blood mononuclear cells (PBMCs) due to smoking. At another candidate gene for smoking, *catechol-O-methyltransferase* (*COMT*), specific CpGs showed differential methylation in smokers *versus* nonsmokers, with a delta beta of approximately 6% at the site with the greatest difference [38]. This study was done in an African-American (AA) population, highlighting the need to replicate findings to establish generalizability across differing ethnic groups.

Extending candidate gene studies to other populations and tissue sources, researchers assessed the impact of maternal smoking on promoter methylation at *brain-derived neurotrophic facto*r (*BDNF*) in adolescent offspring whole blood samples [39], and *cytochrome P450 oxidase 1A1* (*CYP1A1*) in placental samples [40], finding hypomethylation in each case. Murphy and colleagues [41] examined differences in methylation at two loci, the imprinted domain at 11p15.5, expressing paternal *Insulin-like Growth Factor II* (*IGF2*), and maternal *H19*, a noncoding RNA, in smoking exposed *versus* non-exposed infants, finding that infants born to smokers had increased methylation at the *IGF2* differentially methylated region (DMR) as composed to those who never smoked or quit during pregnancy. Finally, given the established link between smoking and cancer, cancer-related candidate genes were studied using exhaled breath condensate in smokers and non-smokers, showing differential patterns of methylation at *ras association domain family 1 isoform A* (*RASSF1A*) related to smoking status [42].

With the advent of the cancer-focused Illumina GoldenGate methylation platform, several groups examined genome-wide differences in methylation patterns in smokers *versus* non-smokers. In lung tissue, Christensen and colleagues [43] found a significant effect of pack years smoked on *human mutL homolog 1* (*MLH1*) and *receptor-interacting serine-threonine kinase 3* (*RIPK3*) methylation, and 138 loci in total with altered methylation in lung tissues of ever *versus* never smokers. Breton and colleagues [44] found differences in global methylation of LINE-1 and validated hypermethylation at two loci (*tyrosine-protein kinase receptor UFO, receptor-type tyrosine-protein phosphatase O*) by confirmatory pyrosequencing in buccal samples of children exposed *versus* non-exposed to smoking during pregnancy.

Subsequently, two array-based platforms were developed which allowed a much broader investigation of differential methylation patterns across the genome: the Illumina 27k and 450k platforms. With the development of these platforms, several loci have emerged as robust indicators of smoking. The first consistent locus to emerge, *coagulation factor II (thrombin) receptor-like 3* (*F2RL3*), was covered by both the 27k and 450k platforms, whereas the second, the *aryl hydrocarbon receptor repressor* (*AHRR*), was only covered by the 450k platform but has been more consistently replicated. Several other loci have been replicated in six or more studies as well, including the 2q37.1 region, 6p21.33 region, *growth factor independent 1 transcription repressor (GFI1)*, and *myosin IG (MYO1G)*. However, as we will demonstrate below, the *AHRR* locus fulfills the greatest number of criteria as specified above as indicators of potential for a robust and flexible epigenetic biomarker of smoking. In total, this review identified seven studies using the 27k platform and a further 23 using the 450k platform. Based on the results of these studies, nine more in-depth investigations of promising loci were identified as part of the third wave of studies. Below, we review loci replicated by 7 or more studies, a cutoff chosen after review of our findings to help limit the scope of discussion. It should also be noted that none of the candidate genes investigated in earlier pyrosequencing have demonstrated replicated associations with smoking in later array-based studies.

### 3.2. F2RL3/cg03636183

F2RL3 (coagulation factor II receptor-like 3), is located on chromosome 19p13.11, and is a member of the proteinase-activated receptor family. The consistent association of F2RL3 with smoking status has attracted interest because of the plausible relationship between coagulation pathways and the cardiovascular risks associated with smoking [45].

At this locus, a single CpG probe has emerged consistently: cg03636183. This review identified 12 studies that included the probe among their list of significant results. Of those, the probe was the most significant finding in three [46,47,48], all of which used the 27k platform for discovery. Although subsequent studies with the 450k platform have not typically identified this probe as the most significantly associated with smoking, the overall finding has replicated in a number of these studies. The probe has been shown to be hypomethylated in adult smokers across a broad age range [27,28,46,47,48,49,50,51,52,53,54]. The probe is associated with smoking in studies in both men and women, men only [28], and women only [27,50,53,54]. The association is present in individuals of African ancestry [27,48], South Asian ancestry [28], and Arab ancestry [55] in addition to European ancestry.

Additionally, although the majority of studies reporting associations with probe cg03636183 have not used other biomarkers to confirm exposure to smoking, at least two used cotinine [45,46]. In addition, several studies controlled for blood cell mixture in their analyses, strengthening their findings [23,24,42,45,47,48,49]. Confirmatory sequencing or spectrometry were done in four of the studies [46,49,50,52] and confirmatory qPCR in one [27].

Unfortunately, the association has not replicated in studies using non-adult age groups (including prenatal exposure), or tissues other than peripheral blood cells. This lack of generalizability represents a limitation when compared to the AHRR locus, as will be discussed below. Another limitation is that the maximum magnitude of the finding (absolute difference in average methylation level or delta beta) is reported to be in the range of 8% to 10%, which may limit its detection in smaller studies. Finally, none of the studies finding the association performed genotype by methylation (GxMeth) analyses to avoid confounding of findings by genotype. This is an important consideration because according to dbSNP, the CpG residue interrogated by cg03636183 is 46 bp from rs773902, a SNP which is in marked population disequilibrium [56].

However, despite this potential problem, the locus may have potential as a candidate clinical biomarker for heart disease. Follow-up studies by Breitling, Zhang and colleagues [45,57,58,59], using bisulfite conversion and spectrometry and DNA from European samples have shown that methylation is strongly associated with mortality from all causes, cardiovascular disease, and cancer [45,57,58,59]. The studies establish dose-effect relationships between methylation and current intensity of smoking, pack-years smoked, and years since quitting, and a dose-effect relationship between methylation level and mortality risk. This relationship also appeared to mediate the relationship between smoking intensity and risk of mortality.

### 3.3. AHRR/cg05575921

*AHRR* is located on chromosome 5p15.33. The gene is a key regulator of the *aryl hydrocarbon recepto*r (*AHR*) pathway which is responsible for the detoxification of toxins such as polyaromatic hydrocarbons and dioxins found in burnt products via the P450 cytochrome system [60]. *AHRR* is a complexly regulated gene, with 5 CpG islands, at least 21 known splice variants and 10 known protein isoforms. The exact relationship between methylation changes at each of these CpG islands to production of these isoforms is not well understood. But increased transcription of AHRR protein serves as a negative feedback loop for the AHR mediated activation of CYP1A1, CYP1A2 and CYP1B1 via competitive inhibition of AHR binding to its cognate nuclear receptor (*aryl hydrocarbon nuclear translocator*; *ARNT*) partner or occupation of AHR DNA binding motifs [60]. Interestingly, the most replicated association with this gene is for a probe located within intron 3 of AHRR, not in a promoter region, a region that contains an enhancer motif whose demethylation is associated with the recruitment of DNA Complex C2 and C3, with the subsequent increase in AHRR mRNA production [49,61].

Early array-based studies using the Illumina 27k platform did not identify any association between smoking and AHRR due to lack of coverage of this area. However, among the twenty studies using the 450k platform to test for associations between a wide variety of smoking exposures across varying ages, tissues, ethnicities, and both sexes, only six failed to demonstrate the association between AHRR methylation and smoking. In 11 of those studies, a single probe, cg05575921, was identified as the most associated, and the probe was the second in another [27] and third in yet another [52]. Although significant associations between smoking and other probes in the AHRR region have been reported in a number of studies, in only one [61] were other AHRR probes ranked more highly.

Among the remaining five array-based studies that did not replicate an association between smoking and cg05575921, two used a distinct cell type (buccal scrapings, fetal lung and placenta) [62,63], one examined newborns exposed in-utero [64], and in another, the probe was associated at *p* < 1 × 10^−4^ but did not reach statistical significance. The remaining study, no probes showed any significant associations (likely due to low power), but among all probes tested, probe cg05575921 was the most significantly associated [29]. Thus, findings of significant association between smoking and hypomethylation of cg05575921 have been the rule rather than the exception.

Further supporting the AHRR locus and probe cg05575921 in particular is the variety of studies showing associations. As with F2RL3, studies have replicated the association in samples including men [28,65], women [27,50,52,53,54,61] and both sexes combined [49,50,51,53,66,67,68,69,70,71,72]. The association is present in smokers as young as age 19 [66] through age 60 and later [48]. The signal is robust across multiple ethnic groups [27,28,54], and different windows of exposure [67,69]. The association has excellent quantitative effects, with a high average delta beta of over 20% in many of the studies of older smokers [28,49,53], and effects of both cumulative pack years smoked and cessation time on methylation observable in former smokers [49,54,66]. Many studies have included additional experiments to bolster the validity of findings at this locus, including confirmatory bisulfite pyrosequencing [49,50,52], measurement of AHRR gene expression [27,50,51,53,54,62], statistical techniques to control for GxMeth effects [66] and for peripheral blood cell mixture [27,28,51,53,54,67,68], and replication of findings in additional samples [49,53].

Since the publication of the above array-based, several follow-up studies have gone on to confirm associations between smoking and AHRR and expand findings, often using sequencing or mass spectrometry. These studies once again confirm methylation changes related to prenatal smoking exposure, as measured by cotinine, both in newborns [69], and 18 months later [70]. Two groups combined several loci at AHRR and other genes to determine if combining signals could lead to an improved instrument for assaying smoking exposure status. Shenker and colleagues [71] found that the combination of four CpGs, including cg05575921, provided the best AUC, whereas Philibert and colleagues [72] found cg05575921 to have excellent predictive properties as a single test, with an AUC of 0.99.

What remains to be determined about cg05575921 to move it toward use as a clinical biomarker are better understanding of rates of baseline methylation and decay across populations and more detailed information about its predictive utility with regard to clinical outcomes above and beyond self-reported smoking status and history, as is the case with F2RL3. However, at this time, even as a simple indicator of the presence or absence of active smoking [72], cg05575921 may be considered a biomarker that is ready for candidate clinical status. In the recent study of Zhang and colleagues [59], cg05575921 was among the top two loci most associated with all-cause, cardiovascular, and cancer mortality. Therefore there is emerging evidence that this locus too may have meaningful clinical applications beyond prediction of smoking status, as does F2RL3.

### 3.4. Other Regions

As shown in Table 2, there were seven other genes or regions at which significant probes were identified in the literature reviewed. In general, delta beta values for these regions and probes were less than that of AHRR/cg05575921, making them less ideal as potential biomarkers. At some loci, such as 2q37.1 CpGs cg05951221, cg2156664, and cg01940273, average delta beta values in the studies reviewed were in approximately the 10%–15% range, indicating some potential as adjunctive biomarkers, however. However, in the study of Philibert and colleagues [72], multi-marker models including all three of these CpGs, as well as AHRR CpGs cg05575921 and cg23576855, did not improve the AUC in receiver operating characteristic (ROC) analyses. In addition, at other loci such as 6p21.33 CpG cg06126421, at least one study reported a delta beta value as high as 23% [28], but this magnitude has not been replicated in other studies. Interestingly, the same CpG was among the top two most associated with all-cause, cardiovascular, and cancer mortality in on recent study, although the delta beta for the CpG was only 13% (current *versus* never smokers) [59].

**Table 2:** Genes with significantly associated CpGs for smoking in seven or more studies.

AHRR/cg05575921	15 studies
F2RL3/cg03636183	13 studies
2q37.1	10 studies
CNTNAP2	10 studies
GFI1	10 studies
MYO1G	9 studies
GPR15	9 studies
6p21.33	8 studies
GNG12	7 studies

Continued investigation of the loci listed in Table 2 is warranted, as other loci may provide the ability to quantify other aspects of smoking behavior, such as cessation time and remote smoking behavior [28,49,50,54,62], and may offer differential sensitivity and specificity in different ethnic groups [28]. In addition, although not the primary purpose of biomarker studies, detection of weaker signals may be useful in elucidating the underlying biology of smoking-associated disease, for example through the use of network and pathway analyses [27].

In summary, epigenetic biomarkers for smoking appear to meet the key criteria for potential successful clinical translation, particularly at the two sites with the most replicated associations, AHRR and F2RL3. Among the studies reviewed above, methodological issues are adequately addressed, including controlling for batch effects, cell mixture, confirming exposure with other biomarkers such as cotinine, controlling for other exposures such as cannabis, and most importantly performing careful phenotyping. The studies above include appropriate sensitivity and specificity analyses, demonstrate dose-response relationships between exposure and methylation, and capture both persistent signatures of past smoking and reversible signatures that indicate cessation time. In addition, these loci demonstrate the ability to predict important clinical outcomes such as mortality. In terms of generalizability, the studies include populations of different ages, ethnicities, sexes, use patterns, and periods of developmental exposure. Of note, findings at AHRR and F2RL3 did not replicate consistently across non-blood tissues, indicating that other loci may be more appropriate if other tissue sources are to be used clinically. However, on the whole, this review found sufficient evidence to recommend development of epigenetic biomarkers for smoking as clinical tools with the potential for tremendous public health impact.

### 3.5. Alcohol

In contrast to smoking, health risks associated with alcohol follow a U shaped curve, with modest drinking conferring lower overall levels of risk to health as compared to complete abstention and heavy drinking [73]. However, in many individuals, drinking becomes problematic, either through contributing to accidental injury, or through negative effects on health associated with chronic, heavy intake. The epidemiological link between heavy alcohol use and increased risk of cancer is well established [74]. Interestingly, in contrast to smoking, alcohol-associated cancer risk appears to decline more slowly over time than smoking-associated cancer risk, with 20 or more years required for the risk of head and neck cancers associated with drinking to equal that of abstainers [75]. Both the longevity of risk associated with heavy alcohol use, and its distinct U shaped curve for risk in relation to use pattern suggest that the underlying epigenetic mechanisms at work are distinct from smoking.

Epidemiologic observations related to the comorbid smoking and drinking risks further suggest that distinct epigenetic mechanisms are at play in alcohol use disorders as compared to smoking. In combination, smoking one pack per day and heavy drinking (over 80 grams per day) act synergistically to increase risk of esophageal cancer by up to 44 times [76]. This consistent epidemiologic finding suggests that smoking and drinking have distinct toxicological mechanisms by which risk of disease is conferred. Similarly, it has been demonstrated that while the risks of cancer and other diseases due to smoking are due not to nicotine but to the cumulative effects of the thousands of toxic compounds found in smoke, the risks due to alcohol appear to be directly related to alcohol concentration and dose, with increasing concentration of alcoholic beverages (hence, less exposure to other compounds), conferring increasing risk [74]. From an epigenetic perspective, these findings have led investigators to pursue focused investigations of both candidate genes and broader investigations using array-based platforms to elucidate the underlying mechanisms at play. Relatedly, potential epigenetic biomarkers for alcohol use disorders are likely to follow distinct patterns from those of smoking, as will be detailed below.

Current biomarkers for alcohol are limited in their utility [9]. Perhaps the best characterized biomarker of alcohol is the measurement of alcohol in serum or breath. However, this type of measurement only detects current consumption and does not differentiate between acute consumption and chronic abuse. Other biomarkers of adverse effects related to alcohol have difficulties with sensitivity and specificity, and are not frequently used in clinical practice as screening methods. Given that the magnitude of costs to health and society related to problem drinking are so large, improved biomarkers are necessary. In particular, early identification of problematic drinking patterns, before behavior becomes entrenched, and the ability to monitor for relapse during long-term treatment are essential tools needed to improve prevention and treatment of this disorder.

In reviewing the literature for studies assessing methylation changes associated with alcohol that have potential to translate into clinical biomarkers, several trends emerge. First, fewer studies have used array-based technologies in alcohol as opposed to smoking (10 found for this review). Second, fewer significant associations have been reported and effect sizes are generally more modest in alcohol as opposed to smoking, with top delta beta values frequently under 10%, leaving fewer loci as potential biomarker candidates. Third, the results of candidate gene studies, as listed above, have generally not replicated in later array-based studies. Fourth, many studies have been done using *in vitro* models, animal models, and post-mortem tissues. These have focused on the relationship between histone modifications and chronic alcohol exposure; findings which are likely further from clinical translation but suggest future avenues for research.

Candidate gene based investigations into the biology of alcohol-related disease have focused in a few key areas. The most common focus is well-established neurotransmitter systems commonly studied in psychiatric disorders such as dopamine, serotonin, and glutamate, their receptors, transporters, and enzymes of degradation. The second most common area of investigation is that of more specialized neurotransmitters such as vasopressin, and orexin. A third common theme is genes related to one carbon metabolism. Fourth and fifth common areas of investigation are genes related to craving addiction, particularly with respect to the endogenous opioid system, or those related to neuronal growth and homeostasis.

This review identified fifteen candidate gene methylation studies ranging across all of the above areas. Loci studied included *alpha-synuclein* [77], *DNA methyltransferase 3b* [78], homocysteine-induced endoplasmic reticulum protein [79], *NMDA receptor subtype 2b* [80], *monoamine oxidase A* [36], the *serotonin transporte*r [81,82,83], the *dopamine transporter* [83,84,85,86], the *H19* and IG differentially methylated regions [87], *vasopressin* and *atrial natriuretic peptide* [88], *proopiomelanocortin* [89], *orexin A* [90], *nerve growth factor* [91], *MeCP2* [83], *leptin* [92], and the *mu opioid receptor* [93]. Studies generally reported small but significant changes in methylation at the above loci. A few loci, however, have had consistently negative results [81,82], failed to replicate previous associations [85,86], or found associations in subgroups only [36].

With the development of array-based methylation profiling, investigators have been able to look more broadly at candidate and genome-wide loci. Four studies using Illumina’s GoldenGate array platform for methylation were found. Two of the studies used the cancer candidate gene focused panel available from the manufacturer [43,94], while two others by the same group [95,96] used custom arrays for alcohol-related candidate genes on the same platform. Although limited by a small sample size of 29, Christensen and colleagues [43] reported 12 hypermethylated CpGs and 20 hypomethylated CpGs at candidate cancer genes, the latter including *HTR1B*. The other study using the cancer candidate gene focused platform found no significant differences due to smoking, and only 5 sites with a delta beta of more than 5%. Interestingly, in a subgroup of alcoholic smokers *versus* their abstinent siblings, a significant difference was found in mean methylation. Among the two studies by the same group using the custom array, positive associations with *HTR3A* were found in one study, though in European Americans only [95], and in the other, no associations with alcohol use alone were found, but there appeared to be an effect of childhood adversity on methylation at two loci (*CHRNA5*, *HTR1B*) in the combined alcoholic and non-alcoholic European American sample [96].

Two studies using Illumina’s 27k platform were found. The first, using a small sample size of 20 alcohol dependent cases *versus* controls [97] reported differential methylation occurring within individuals between two time points at 252 genes in controls, 200 in cases, and 3 in both. Unfortunately, the published article does not provide the specific gene list or corresponding *P* values (noting only that the reported genes had *p* values < 0.01). The second study [98] had a larger sample size of 128 participants, and reported significant associations at 1710 CpG sites (*p* < 0.005 after Benjamini-Hochberg correction and delta beta ≥ 17%), also reporting that all of the 50 sites were hypomethylated. The authors report the differentially methylated loci to include two alcohol dehydrogenases, one aldehyde dehydrogenase, and CYP2A13, and five loci with delta beta values over 40%, including *C8orf4*, *HCRTR1*, *FLJ38379*, *HSA277841,* and *TSC2*, but do not include a list of methylation values, *p* values for association in the publication or supplemental materials.

Four studies using Illumina’s 450k platform were found. Of these, one was previously reviewed [61], and reported as a non-primary outcome a nominal association of two probes with drinking after controlling for smoking. A second found no differences in methylation at CpG sites between groups stratified by alcohol intake, but did find one significant probe when pooling moderate and heavy drinkers *versus* abstainers, and found further evidence of association with the BLCAP region using an 11-probe sliding window technique [99]. A third study [100] using AD-discordant siblings found 865 hypomethylated and 716 hypermethylated sites, as defined by a DiffScore of 20 or greater (log transformation of *P* value, corresponding to 0.05 < *p* < 0.01 according to Illumina materials), with GABRP among the top thirty hypermethylated sites.

The last study by Philibert and colleagues [101] was designed to overcome limitations of some of the above studies. Specifically, instead of looking at the trait of alcohol dependence in the presence or absence of recent alcohol use, it purposively selected active, heavy alcohol consumers entering and exiting treatment for alcohol use disorders *versus* community controls who were selected from an environment in which alcohol use was discouraged whose self-reports were confirmed by objective biomarkers for smoking and cannabis use. Methylation signatures on treatment entry were compared with those of controls and with those of the same alcohol-dependent individuals at 4 weeks following treatment entry. Although significant changes within individuals in the four weeks following treatment entry were not detected, 8626 probes were found to be differentially methylated between cases and controls after conservative Bonferroni correction, with top delta beta values generally less than 10%. Of note, the largest delta beta of approximately 15% was seen for *GFI1*, a gene reported in a number of smoking studies (see above), suggesting possible confounding at this locus.

The largest methylome-wide study to date was the recent study of Clark and colleagues [102], examining over 600 individuals at approximately 27 million CpG sites in approximately 4 million CpG “blocks” using a novel methyl-CpG-binding domain (MBD) protein-based sequencing technology as well as a genome-wide association study using the Affymetrix 6.0 chip. Unfortunately, despite the innovative technique employed to interrogate the methylome, phenotyping was a relative weakness of the study, which employed a binary question asking if participants had ever consumed alcohol regularly *versus* never consumed alcohol regularly. In their discovery sample, 94% of subjects answered affirmatively, as did 93% of those in the larger replication sample of 730 subjects. Although Clark and colleagues reported significantly different methylation at 33 “blocks” or DMRs at a FDR threshold of q < 0.1, there is no overlap with these blocks and the top CpGs in the second-largest array-based study of Philibert and colleagues [101] or that of Zhao and colleagues [100].

Lastly, although of limited usefulness as a clinical biomarker due to low specificity, six studies assessing global methylation levels were found. The first [103], found striking differences in global methylation, with hypermethylation of +10% as measured in HpaII/MspI digestion fragments and cytosine extension. A later study using the same technique found smaller (+7%) difference of methylation in alcoholics as compared to controls [79]. However follow-up studies by others [31,104] failed to replicate this difference when using a different method, bisulfite pyrosequencing of global LINE-1 elements, although a modest difference (0.2%) in Alu methylation was found by the same methods in one study [104]. It is likely that this difference is due in part to different techniques used. A fifth group [105] found no effect of stratified alcohol intake on global methylation, but did report a weak interaction between alcohol and folate intake on methylation. Postmortem studies in human brain tissue have shown mixed results regarding global methylation, with one showing global hypomethylation as measured by Qpcr [106], but another showing no significant differences [107]. Most recently, Semmler and colleagues also recently reported global hypermethylation in lymphocytes was correlated with alcohol consumption and smoking on treatment entry for alcohol detoxification [108].

There are numerous limitations apparent on review of the above literature, both in terms of quality and quantity of studies. The primary weakness is the direct lack of any replicated finding thus far for any pattern of alcohol use. However, promisingly, recently the top markers for alcohol consumption from Philibert (2014) have been shown to demonstrate the classic “U-shaped” curve effect on survival in a large community sample (*n* = 656) [109]. In general, candidate gene associations have not replicated well in array-based studies, although this may be in part due to lack of coverage. The literature using 27k data is unfortunately limited by lack of reporting of full findings and methods. Among the two more well-powered studies using Illumina’s 450k array, one used a non-primary source of DNA [99], transformed lymphoblasts, and suffered from potential weakness in characterizing alcohol use patterns (self-report over the last 6 months). The second study [101] addressed those issues and found a much larger number of associations, suggesting that some of these findings, if replicated, could point toward the development of clinical epigenetic biomarkers. It is interesting that the study of Philibert and colleagues [101], did not replicate the associations or large delta beta values (one over 50%, 23 others over 20%) of the report of Zhao and colleagues [100], suggesting these differences may be due to study design or underlying population differences. Finally, in addition to the inconsistency of results, global methylation is unlikely to translate as a biomarker for alcohol use due to its lack of specificity.

Going forward, more and larger studies will be needed to determine if the very preliminary findings above can be replicated, as have been done for smoking. Building on the experience from the more successful studies of smoking in which state dependence of methylation changes are evident, studies of alcohol use phenotypes will likely benefit from careful attention to the effects of periods of abstinence on methylation signatures.

### 3.6. Cannabis

Cannabis is the most widely used illegal drug [4] in the United States. Cannabis abuse and dependence are also commonly comorbid with other substance use disorders, particularly smoking [110]. Cannabis use in the United States has increased in recent years, particularly among children and adolescents, raising concern about the effects of exposure on the developing brain. Recently, states such as Colorado have also taken steps to decriminalize cannabis, leading to concerns about the consequences of increased use in children and adolescents as well as adults [111].

The psychoactive components of cannabis are the cannabinoids, including delta-(9)-tetrahydrocannabinol (THC). Cannabinoid receptors CB1 and CB2 and their endogenous agonists such as anandamide have been discovered in recent years. THC and other exogenous cannabinoids can be measured in a number of tissues, including hair, saliva, blood, and, most commonly, urine [112].

In reviewing the available literature for potential epigenetic biomarkers of cannabis use, there were no array-based studies and only two studies using peripherally available sources of DNA in human subjects, both candidate gene studies by the same group.

Noting emerging interest in the interaction between cannabinoids and orexins, a class of molecules involved in regulation of appetite, arousal, and energy regulation, Rotter and colleagues [113] examined a differences in *Orexin A* expression between cannabis dependent individuals, (tobacco) cigarette smokers, and non-smoking controls. Expression was measured by quantitative PCR in peripheral lymphocytes, and promoter methylation measured by methylation specific digestion and subsequent quantitative PCR. Cotinine, THC and metabolites of THC were not measured for comparison, nor were analyses adjusted for blood cell composition. Significant differences in *Orexin A* expression were found between all three groups. There was no difference found in mean *Orexin A* promoter methylation between groups, but their method precluded measurement of individual CpG methylation status to examine for differences between groups. Despite the lack of overall difference in methylation found, the differences in expression suggest that further investigation of this locus as a potential biomarker is warranted.

In a parallel study, using the same subjects and general methods, Rotter and colleagues [114] also measured CB1 and CB2 expression and CB1 promoter methylation. CB1 expression was significantly different between all three groups, as was CB1 promoter methylation, with cannabis dependent subjects having the highest level of methylation (89%), followed by cigarette smokers (84.4%) and non-smokers (62.5%). CB1 promoter methylation was negatively correlated with expression in all three groups as a whole. CB1 expression levels also correlated significantly with clinical variables including craving. As in the previous study, cotinine and cannabinoids were not measured for comparison, analyses were not adjusted for blood cell composition, and site-specific CpG methylation levels could not be assessed. Despite this, these results suggest CB1 promoter methylation and gene expression as additional potential biomarkers for THC dependence.

Finally, although with less potential for clinical translation, one study using human fetal brain tissue was reviewed. DiNieri and colleagues [115] examined the effects of prenatal cannabis exposure in postmortem human fetal subjects and in rats prenatally exposed to cannabis. In the fetal subjects, *in situ* hybridization histochemistry was used to measure expression of *DRD1*, *DRD2*, *PENK*, and *PDYN i*n the nucleus accumbens (NAc) in cannabis-exposed cases and controls. Cannabis exposure was confirmed by maternal self-report and/or urine THC and/or fetal meconium THC. Of the four genes studied, only *DRD2* expression was significantly correlated with cannabis exposure, with decreased expression levels detected. In the rat model, increased *2meH3K9* and decreased *3meH3K4* and *RNA polymerase II* expression were found at the *DRD2* locus, as well as decreased *DRD2* expression.

In summary, the literature on potential epigenetic biomarkers for cannabis use disorders and exposure are extremely limited and, in contrast to smoking, there do not appear to be any loci which meet the criteria outlined above for potential clinical translation. There was a single significant association found in one study between *CB1* promoter methylation and cannabis dependence. Although the magnitude of change in methylation at this locus between cannabis-dependent subjects and non-smokers was appreciable at 26%, the difference between cannabis-dependent subjects and cigarette smokers was much more modest at less than 5%, casting doubt on the potential applicability of this finding. Nonetheless, there is a great deal of potential for the development of epigenetic biomarkers for cannabis use simply because no studies have been done using the larger array-based platforms to look more broadly for associations. There is some possibility that unique signatures may be found, particularly if studies are done with careful clinical characterization and confirmation of exposure via existing biomarkers such as ELISA for THC metabolites. It remains to be seen if these studies will be able to sufficiently distinguish a cannabis-related signature from that of tobacco smokers, which is a critical issue given the high comorbidity of tobacco smoking among cannabis users. The other design considerations, which include applicability across populations, the ability to find an epigenetic signature in accessible peripheral tissues, sensitivity, and specificity, as well as cost, all apply to this area as well and will need to be taken into consideration.

### 3.7. Opioids

Heroin and other opioids are highly reinforcing substances, which can cause substantial harm to individuals’ health over time. According to the Substance Abuse and Mental Health Services Administration (SAMHSA) [4], over two million individuals in the United States were dependent on or abused heroin and other pain relievers in 2010, with over 1 million of those reporting for treatment in the last year. Despite the widespread availability of pharmacologic and behavioral interventions for opioid addictions, significant challenges remain in tackling this public health problem. A key related public health issue is that chronic administration of opioids often takes place through intravenous injection, placing users at markedly higher risks for transmission of HIV, hepatitis C virus, and other infections, which collectively dramatically add to the health burden associated with this disorder. Additionally, dependence on prescription opiates being administered for acute or chronic pain is an emerging area of concern. Current biomarkers for opioid dependent are limited. The most commonly used screening tests in clinical practice are urine-based and have a detection limit of approximately 3–4 days for most opioids [116].

Opioids exert their psychoactive effects through opioid receptors in the brain. Of the three opioid receptor genes, *OPRM1*, *OPRD1*, and *OPRK1*, which encode the mu, delta, and kappa opioid receptors, *OPRM1* is the most studied with respect to opioid addiction, as it is the main site of action of commonly used opioids such as heroin, morphine, and methadone [117]. It should also be noted that behavioral reinforcement via endogenous opioids plays a role in the formation and maintenance of many other types of substance use disorders, such as alcoholism [93].

In this review, four candidate gene studies assessing methylation patterns at CpG islands associated with the OPRM1 promoter region were identified. Two studies by the same group characterized associations between specific CpGs in Caucasians [118] and subsequently in Hispanics and African-Americans maintained on methadone [119], finding associations differed between ethnicities. Overall, differences were fairly small, with the largest reported as 7.1% at CpG −25 in Hispanic former heroin addicts *versus* controls, as compared to the largest being 5.6% at CpG +12 in African-Americans, who had significantly higher baseline rates of methylation across the CpG island as compared to Caucasians and Hispanics. Of note, the former study used both lymphocytes and whole blood, whereas in the latter lymphocytes only were used.

In addition to the peripheral blood studies mentioned above, one study compared *OPRM1* promoter methylation in whole blood and sperm among male heroin addicts. Significant hypermethylation was found at seven CpG sites in blood from addicts *versus* controls, whereas only a single CpG showed hypermethylation in sperm. Interestingly, overall methylation was significantly lower in sperm compared to blood.

The final study consisted of a mix of methadone maintained former heroin addicts, healthy controls, opioid-treated chronic pain patients, and non-opioid treated chronic pain patients. DNA was extracted from whole blood and both global methylation at LINE-1 repeats and local methylation patterns at 22 CpG loci in the OPRM1 promoter region were analyzed with respect to the above groups. Increased methylation at CpG +126 and global LINE-1 were seen in the methadone patients as compared with controls, and additional analyses including smoking as a covariate confirmed hypermethylation at LINE-1 but not CpG +126. Similarly, in the opioid-treated pain patients *versus* non-opioid-treated pain patients, global methylation at LINE-1 was significantly higher, as was local methylation at CpG +126. LINE-1 methylation but not OPRM1 methylation at any CpG was also higher in the methadone patients as compared to opioid-prescribed pain patients. Interestingly, LINE-1 methylation but not OPRM1 methylation in the chronic pain patients was significantly correlated with pain intensity, but not in the methadone patients.

No studies of human subjects with opioid use disorders assessing methylation signatures via large-scale arrays such as the 27k or 450k platform were found. In summary, therefore, the literature on potential epigenetic biomarkers for opioid use disorders do not indicate any findings that are ready for potential clinical translation, for the reasons outlined above, despite the urgent need for improved clinical tools.

### 3.8. Psychostimulants

Psychostimulants is a loose category of substances that include cocaine and amphetamines which produce temporary increases in alertness or physical activity. SAMHSA estimates place the estimated number of people addicted to cocaine in the United States at over one million as of 2010 [4]. Cocaine and amphetamines are potent sympathomimetic drugs which increase synaptic concentrations of dopamine, norepinephrine, and to a lesser extent, serotonin through multiple mechanisms. The potent reinforcing effects of these medications are thought to be related, in part, to epigenetic changes that can occur rapidly after initial administration. Recent reviews have outlined the spectrum of epigenetic findings, primarily using animal models and postmortem brain tissue, which have implicated a several distinct epigenetic changes occurring with exposure to psychostimulants, including histone modifications [120], as well as changes in methylation at specific genes [20] and changes in noncoding RNA expression [121]. It is also important to note that several recent studies, including seminal work by Feng, Nestler, and colleagues [122,123] have investigated the role of Tet1 and 5-hydroxymethylcytosine (5-hmc) cocaine addiction using animal models. These studies are important because demethylation, in addition to methylation, plays a role in gene regulation in substance use disorders. Unfortunately, these literature reviews and our own are limited by the lack of studies using peripheral tissues from living, human subjects in order to ascertain potential epigenetic biomarkers for psychostimulant use disorders. Review of these articles and our PubMed search did not find any studies using Illumina arrays that met those criteria. Therefore, at this time, we conclude that there is no epigenetic finding with clear potential for clinical translation for the assessment of psychostimulant use disorders, though there is certainly an important public health need for the development of these.

## 4. Discussion

Our review of the literature demonstrates that the search for epigenetic biomarkers for substance use disorders is progressing and in certain cases, shows considerable promise. The search for smoking biomarkers is the most advanced, with multiple sites showing replication and potential for translation to clinically validated biomarkers. The literature for alcohol is much more limited with a few more recent studies using Illumina’s methylation array platforms finding potential loci reflecting alcohol use patterns, albeit with more subtle signals as compared to smoking. Finally, for other substances such as cannabis, opioids, and psychostimulants, the evidence is still more limited, with either a few candidate gene studies (opioids, cannabis), or in the case of psychostimulants, no studies using peripherally available tissue from living human subjects. Clearly, the way forward is to conduct larger studies with careful phenotyping, using a variety of study designs and a variety of populations in order to capture more robust signals. Of note, supplemental network and pathway analyses may be useful even in the absence of highly significant findings in order to elucidate the underlying biology of chronic substance exposure if they point toward relevant underlying biological pathways. Below, we highlight several guidelines to consider when designing such studies, based on the results of our review, in order to maximize the chances of finding epigenetic biomarkers for substance use disorders, while also addressing issues of replicability, generalizability, and cost. We will also address challenges in the design and interpretation of such studies, and acknowledge important limitations of the current literature.

To address the issue of replicability of findings, future studies will need to be more standardized to maximize the chance of replication, including best practices for handling data. Relevant study design issues include batch effects, normalization of signals, analysis of confounding variables, ascertainment bias, sample processing, genotype by methylation confounding (GxMeth) and controlling for cell mixture. Fortunately good techniques exist to address these. The inclusion of other biochemical markers of exposure such as urine or serum cotinine is also recommended.

In order to address issues of generalizability across populations, more studies using a variety of populations and tissue types are needed. It is likely to be increasingly challenging to find early, subtle signs of exposure to illicit substances due to their lower rates of use and users’ wish to avoid detection. For some substances such as prescription psychostimulants, it may be helpful to obtain pilot data from children and adolescents chronically being prescribed these medications, as there are likely to be fewer confounding exposures at earlier ages.

To address the issue of cost, less expensive methods of assessing methylation patterns at loci that have been well-validated should be investigated. For example, quantitative PCR may be quite inexpensive and suitable for well-characterized loci such as AHRR. However, as the expense of methylome-wide arrays continues to fall, routine screening at a large number of loci may become more commonplace. If so, best practices for interpretation will be needed, as experience with rise of genetic testing marketed to individual consumers suggests there is great potential for misinterpretation of findings. It is also possible that the technology to assay other epigenetic biomarkers such as histone modifications may become less expensive and future studies may more routinely use these technologies to add to our understanding of the total picture of epigenetic regulation in the setting of substance use.

Some potential challenges lie in the way of these goals, relating to the diverse nature of substance use disorders themselves, comorbid use patterns, and limitations of current technology. As noted above, smoking is unique in the number and type of toxic exposures it causes to human cells, which may account for our ability to detect to downstream epigenetic changes. In contrast, other substances such as cocaine may cause dramatic changes in behavioral patterns, both acutely and chronically, as well as cause physiologic dependence, but may not cause a robust epigenetic signature detectable by current technology. If so, this may be due to the fact that the mechanism of action of many such substances is mediated by tissue-specific receptors, as opposed to the toxic aromatic compounds found in burnt tobacco products, or alcohol, which is itself a solvent molecule able to penetrate cell membranes throughout the body. However, it is possible that other kinds of epigenetic changes such as histone modification, for which current assays are less advanced, will become detectable as technology improves.

An additional challenge that has not been met by the current literature will be identifying use patterns for specific substance in the presence of poly-drug use. Although some of the studies mentioned above were able to identify the presence of poly-drug use through objective measures such as cotinine and hydroxyl-THC ELISA [101], it remains unclear how best to control for the presence of poly-drug use in epigenetic analyses targeted toward biomarker development. Specifically, with respect to smoking, the high rate of comorbid smoking among users of other substances and the relatively strong epigenetic signature of smoking may obscure detection of signals from other compounds. To overcome these challenges, careful study design using objective markers of use are essential. If methylation signatures of substances such as cocaine and opioids are relatively subtle, misclassification of use patterns in case *versus* control designs has the potential to seriously damage the power of such studies. From a bioinformatics perspective, it is also likely that more sophisticated algorithms will need to be developed to carefully distinguish the epigenetic signatures of different poly-drug use patterns, for example cannabis smoking in individuals who also smoke tobacco.

Lastly, two important limitations of the microarray-based studies reviewed above should be noted. First, some Illumina probes (up to 25% in some studies) have been identified as cross-reactive and non-specific [124,125,126]. Therefore, measurements using these probes will be influenced by methylation levels at multiple sites, which may cause false negative as well as false positives. A second limitation of current array-based technology is that it measures only a small proportion of CpGs in the genome. Future approaches such as whole-genome bisulfite sequencing may allow a much more thorough examination of epigenetic changes across the genome and could provide significantly improved biomarkers for substance use disorders, particularly those mentioned above for which the current technology has provided minimal results.

## 5. Conclusions

In summary, we have shown that the science of epigenetic biomarkers for substance use disorders is still in its infancy. For smoking, the substance which causes the greatest morbidity and mortality worldwide, several promising loci found using array-based technology have replicated across multiple studies and have potential for clinical translation. For alcohol, fewer studies have been done and epigenetic signatures appear to be more subtle, but there is potential for translation if recent findings are replicated. For other substance use disorders, despite active investigations in the areas of candidate gene studies and animal models, minimal study using array-based technology has been done and there are no loci with immediate potential for clinical translation.

For smoking, the next step will be to translate these findings into clinical tools that can then be studied in interventions aimed at identifying smoking behavior, monitoring smoking cessation during and after treatment, and assessing risk for related health complications. For alcohol, further study and replication are needed to identify which loci should be developed for such clinical tools, but translation may occur in the near future. Other substance use disorders will require a significant increase in study and replication of any findings before translation can be considered.

To that purpose, several guidelines, challenges, and limitations to consider in study design have been outlined above to help guide researchers in the pursuit of improved epigenetic biomarkers for substance use disorders. Substance use disorders continue to cause significant morbidity and mortality on a worldwide basis, and thus more study is urgently needed to provide clinicians with the tools to detect and treat these disorders.
